# Analytical Performance and Clinical Evaluation of a Fully Automated Multiplex RT-qPCR Platform for Rapid Detection of Central Nervous System Pathogens

**DOI:** 10.3390/microorganisms14091927

**Published:** 2026-09-01

**Authors:** Mesut Yilmaz, Naim Mahroum

**Affiliations:** Department of Infectious Diseases and Clinical Microbiology, International School of Medicine, Istanbul Medipol University, 34810 Beykoz, Istanbul, Türkiye; myilmaz@medipol.edu.tr

**Keywords:** meningitis, encephalitis, multiplex RT-qPCR, limit of detection, accuracy, precision, cross-reactivity

## Abstract

Meningitis and encephalitis necessitate rapid pathogen identification to guide therapy, as conventional methods are time-consuming. This study evaluated both the wet-lab analytical performance and the clinical performance evaluation of the Bioeksen Meningitis/Encephalitis Panel (BS-MEP) integrated onto the fully automated, high-throughput Sigmoida Lab platform. Analytical limits of detection (LoD) were defined via Probit analysis (95% threshold) by spiking negative cerebrospinal fluid (CSF) matrices. Target detection was verified using characterized reference materials for all 14 analytes, and in silico primer/probe coverage was assessed against taxon-specific sequence databases. Cross-reactivity was evaluated using high-prevalence non-target organisms. Clinical performance was evaluated retrospectively using 500 archived, anonymized CSF specimens from a single-center repository, with analyte-specific classifications compared with prespecified routine comparator methods. The automated platform provided an 80-min sample-to-result turnaround for up to 23 samples. Verified LoDs ranged from 472 to 2118 genome copies/mL. All inclusivity strains were successfully detected in 5/5 replicates. In silico coverage exceeded 98% combined, and zero wet-lab cross-reactivity was observed. Precision coefficients of variation (CVs) were consistently ≤1.38%. In the clinical evaluation, the pooled clinical agreement was high, with PPA ranging from 90.9% to 100% and NPA of 100% across the evaluated analytes. Analyte-level clinical sensitivity ranged from 90.9% to 100%, with zero false-positive results across all targets. The fully automated molecular system demonstrates excellent analytical robustness and strong clinical agreement for syndromic detection of major CNS pathogens. The automated workflow combines broad pathogen coverage with an approximately 80-min sample-to-result turnaround and warrants further prospective evaluation in routine clinical settings.

## 1. Introduction

Central nervous system (CNS) infectious diseases such as meningitis and encephalitis are severe diseases associated with rapid neurological decline, death or long-term sequelae [1,2]. While meningitis refers to inflammation of the meninges surrounding the brain and spinal cord, encephalitis is characterized by inflammation of the brain parenchyma itself. Clinically, patients typically present with combinations of fever, headache, neck stiffness, altered mental status, seizures, or focal neurological deficits, although considerable overlap between the two syndromes frequently occurs. Because these infections are caused by various bacterial, viral and fungal pathogens, timely identification of the causative agent is crucial to guide targeted antimicrobial therapy and prevent devastating outcomes [3,4]. Traditional culture methods, including Gram stain, culture, antigen testing, and single-target PCR, remain cornerstones of diagnosis but suffer from critical limitations, particularly for viral pathogens or patients who have already received antibiotics [5]. In contrast, multiplex nucleic acid tests offer simultaneous detection of multiple agents, potentially reducing time to diagnosis, permitting prompt management and therefore decreasing the risk of complications [6,7]. However, implementation remains constrained by workflow trade-offs. High-capacity centralized molecular systems often depend on batching and introduce longer turnaround times, whereas rapid near-patient platforms usually offer lower throughput and higher per-test costs. Laboratories are thus forced to continuously balance clinical speed against operational capacity.

In recent years, syndromic multiplex molecular panels have transformed the diagnostic approach to suspected CNS infections by enabling simultaneous detection of multiple bacterial, viral, and fungal pathogens from a single cerebrospinal fluid (CSF) specimen [8]. Compared with conventional culture-based methods or sequential singleplex PCR assays, these platforms substantially reduce diagnostic turnaround time and improve early pathogen identification, particularly in patients who have already received empirical antimicrobial therapy. Commercially available systems, including the BioFire^®^ FilmArray^®^ Meningitis/Encephalitis Panel, QIAstat-Dx Meningitis/Encephalitis Panel, and other multiplex PCR platforms, have demonstrated high diagnostic accuracy and significant clinical impact in reducing unnecessary antimicrobial exposure and supporting earlier targeted treatment [9,10]. Nevertheless, important operational challenges remain. Many rapid systems are designed for low-throughput, near-patient testing, whereas high-capacity centralized molecular laboratories frequently rely on batched workflows that inevitably delay result reporting. Consequently, there remains a need for automated molecular platforms that combine broad pathogen coverage with rapid turnaround times while maintaining the throughput required for routine clinical microbiology laboratories.

The Sigmoida Lab platform combined with the Bioeksen Meningitis/Encephalitis Panel (BS-MEP) was developed to bridge this gap. The BS-MEP is a multiplex real-time reverse transcription PCR (RT-qPCR) assay designed for the qualitative detection of 14 clinically significant pathogens in human CSF: *Cryptococcus neoformans*/*gattii* complex, *Listeria monocytogenes*, *Neisseria meningitidis*, *Streptococcus pneumoniae*, *Haemophilus influenzae*, *Streptococcus agalactiae*, *Escherichia coli* K1, cytomegalovirus (CMV), enterovirus, human parechovirus, varicella-zoster virus (VZV), herpes simplex virus 1 (HSV-1), herpes simplex virus 2 (HSV-2), and human herpesvirus 6 (HHV-6). Consistent with our previous work, we have demonstrated the excellent clinical utility of this syndromic chemistry in an earlier 17-pathogen formulation (which included HHV-7 and HHV-8 and *Cryptococcus* complex counted as two for *C. neoformans* and *C. gattii* in the clinically used panel) [8].

The present study focuses on the streamlined 14-target panel integrated within a next-generation automated workflow. We aimed to establish its full scientific validity by determining the precise mathematical limits of detection (LoD) for each analyte, evaluating analytical inclusivity and in silico primer/probe coverage, confirming analytical specificity via cross-reactivity testing, and validating overall repeatability and reproducibility. Crucially, we expand upon prior laboratory validation by performing a large-scale clinical agreement study using 500 archived, single-center clinical specimens to inform clinicians and microbiologists about the operational reliability and performance metrics of this automated system in routine practice.

## 2. Materials and Methods

### 2.1. Device and Kit Composition

The panel comprises pre-capped four-well PCR strips containing concentrated primers, hydrolysis probes, reverse transcriptase, a hot-start DNA polymerase, dNTPs and quaternary ammonium compounds. A synthetic nucleic acid template serving as a reactive control is included in one well.

Unlike manual setups, processing is entirely managed by the Sigmoida Lab benchtop instrument. This system provides fully automated end-to-end testing, including barcode-based sample identification, automated tube decapping/recapping, robotic magnetic bead-based nucleic acid extraction, real-time PCR amplification, and automated result interpretation via integrated Sigmoida software v8.6REV.144 (Bioeksen AR GE Teknolojileri A.Ş., Istanbul, Türkiye). For CNS testing, the system accommodates continuous loading, allowing additional specimens to be added at approximately 30-min intervals without requiring batching, and yields a sample-to-result analytical turnaround time of approximately 80 min for up to 23 samples per run.

### 2.2. Test Principle

For the archived specimens included in this study, a residual CSF volume of at least 500 µL was required for testing. Once the raw CSF sample tube is loaded into the instrument, automated extraction takes place. The subsequent one-step multiplex RT-qPCR converts targeted viral RNA to cDNA at 52 °C, followed by the co-amplification of cDNA and genomic DNA targets through 30 thermal cycles.

The integrated optical cycler measures fluorescence across multiple channels after each cycle to record real-time amplification data. Positive results required successful amplification of the internal control together with target-specific amplification crossing predefined fluorescence thresholds according to the manufacturer’s validated interpretation algorithm. Samples failing internal control quality criteria were automatically classified as invalid and required repeat testing. The proprietary software subsequently reports qualitative positive, negative, or invalid results [11,12].

The molecular targets, nucleic acid types, target genes or genomic regions, and expected amplicon lengths for all 14 analytes included in the BS-MEP assay are summarized in Table 1.

### 2.3. Reference Material

Analytical validation was performed using analyte-specific NucleoRef Standard Cell Materials supplied by Oligo X Biyoteknoloji A.Ş. (Istanbul, Türkiye). Each material was provided as a single vial containing 10^5^ cells in 200 µL of nuclease-free phosphate-buffered saline. The materials were inactivated by mild heat treatment, and their assigned quantities were determined using the QIAcuity Digital PCR System (QIAGEN, Hilden, Germany). The products were manufactured in a facility operating under an ISO 13485 [12]-certified quality management system and were supplied for research use in the evaluation of nucleic acid extraction and detection procedures, including LoD assessment and monitoring of assay, reagent-lot, and operator variation.

The same analyte-specific reference materials were used for LoD determination, five-replicate wet-lab target-detection verification, precision, and analytical accuracy. Materials were stored at −18 to −20 °C, repeated freeze–thaw cycles were avoided, and working aliquots were prepared according to the supplier’s instructions. The organism names, catalog numbers, material characteristics, and analytical studies in which each material was used are provided in Supplementary Table S1.

### 2.4. Automated Result Interpretation

Amplification data were interpreted automatically by Sigmoida software using the target-specific fluorescence-threshold and cutoff values defined in the current BS-MEP instructions for use. A target was classified as positive when its amplification curve demonstrated an acceptable sigmoidal profile, crossed the predefined fluorescence threshold, and fulfilled the corresponding target-specific cutoff criterion. A target was classified as negative when no valid target-specific amplification meeting these criteria was observed and the internal and reactive controls met the predefined validity requirements.

A result was classified as invalid when the required control criteria were not fulfilled, when amplification did not conform to an acceptable sigmoidal profile, or when the predefined run-validity criteria were not met. Assay-specific cutoff and fluorescence-threshold values were applied exactly as defined in the current instructions for use and embedded in the validated software rules; these parameters were not manually modified during routine analysis.

### 2.5. Limit of Detection

Limit of detection (LoD) determinations followed APHL and Westgard guidance [13,14] defined as the lowest analyte concentration associated with a model-estimated detection probability of at least 95% [15]. Negative clinical CSF matrices were spiked with the analyte-specific NucleoRef Standard Cell Materials supplied by Oligo X Biyoteknoloji A.Ş. An initial concentration series ranging from 100 to 8100 genome copies/mL was tested in quadruplicate to identify the lowest concentration yielding complete detection. The candidate concentration, designated X, together with 2X, X/2, X/3, and X/4 concentrations, was subsequently tested in 24 replicates per concentration.

Replicate outcomes were coded as detected or not detected and analyzed using a binomial generalized linear model with a Probit link function. The final LoD95 was obtained by inverse prediction as the concentration corresponding to a model-estimated detection probability of 0.95. The manufacturer-claimed concentration was separately verified using 24 independent replicates. The hit rate was calculated as the number of positive replicates divided by the total number tested, and at least 23 positive results among 24 replicates were required for acceptance.

### 2.6. Wet-Lab Target-Detection Verification

Wet-lab target detection was verified using the same characterized analyte-specific reference materials used in the LoD study. Each material was spiked into a negative CSF matrix at its established LoD concentration and tested in five replicates. Complete detection in 5/5 replicates was required for acceptance. Mean Cq values and sample standard deviations were calculated for each target [11].

Because the same reference materials were used in the LoD and five-replicate experiments, this experiment was interpreted as verification of consistent target detection at the established LoD rather than as an independent strain-level inclusivity study. Broader genotype-level inclusivity was evaluated separately through the in silico sequence analysis.

### 2.7. Sequence Retrieval and Dataset Curation

For viral targets, nucleotide sequences were retrieved from the NCBI Virus database using Homo sapiens as the host filter and “complete” as the nucleotide-completeness criterion. For bacterial and fungal targets, the complete nucleotide sequence of the corresponding assay target gene or genomic region was used as the query in NCBI nucleotide BLASTN 2.17.0+ searches [16].

Bacterial and fungal records were retained when query coverage and percentage identity were both between 90% and 100%, the record was taxonomically assigned to the relevant target organism, and the corresponding target region was complete. No restriction was applied according to specimen-collection date.

Duplicate records were consolidated according to accession number so that each accession was represented only once. Records with incomplete target regions, ambiguous taxonomic assignment, insufficient query coverage, or insufficient sequence identity were excluded. The database source, download date, initial filtering criteria, and final number of unique sequences evaluated for each pathogen are reported in Supplementary Table S2.

### 2.8. Oligonucleotide Alignment and Mismatch Analysis

Bioinformatic analyses were performed in R version 4.3.0 using Biostrings version 2.72.1, DECIPHER version 2.0.2, and msa version 3.19. Taxon-specific sequence collections were imported as nucleotide sequence sets and reviewed using multiple-sequence alignment. Each forward primer, reverse primer, and hydrolysis probe was independently compared with the corresponding target sequences using pairwise overlap alignment. Where more than one approved oligonucleotide variant was available for a target, the variant producing the highest alignment score for each sequence was retained.

Mismatch numbers, percentage identity, and mismatch positions were determined using the nmismatch, pid with the PID2 method, and mismatchTable functions, respectively. Each unique sequence was classified as containing zero, one, two, or more than two mismatches for each forward primer, reverse primer, and probe [17,18].

For each oligonucleotide, coverage at the 2-mismatch threshold was calculated as:

Coverage (%) = 100 × (number of unique sequences with no more than two mismatches/total number of unique sequences analyzed).

where assay-level coverage was reported, a sequence was considered covered only when an approved forward-primer variant, reverse-primer variant, and probe each satisfied the predefined ≤2-mismatch criterion for the same target sequence. A simple arithmetic average of the three oligonucleotide percentages was not used.

Allowing up to two mismatches was used as a predefined conservative sequence-screening criterion and not as a direct prediction of amplification success or failure. Wet-lab analytical performance was evaluated independently.

### 2.9. Cross-Reactivity (Analytical Specificity)

Analytical specificity was evaluated via wet-lab exclusivity testing against high-prevalence non-target environmental or infectious organisms likely to be present in CSF specimens. A panel list including *Candida albicans*, *Candida glabrata*, *Candida krusei*, *Cryptococcus albidus*, *Cryptococcus laurentii*, *Saccharomyces cerevisiae*, Epstein–Barr virus, various non-target viridans group *Streptococcus* species, *Citrobacter freundii*, *Corynebacterium striatum*, *Enterobacter aerogenes*, non-K1 *Escherichia coli*, and *Haemophilus ducreyi* was prepared at 10^5^ genome copies/mL and tested in triplicate. Target analytes were required to amplify only with their corresponding oligonucleotide sets, while non-target species were required to yield entirely negative results across all channels. No cross-reactivity (all negative) was accepted as confirmation of analytical specificity [11,17,18].

### 2.10. Precision and Analytical Accuracy

Precision was evaluated under predefined repeatability and reproducibility conditions using negative CSF matrices spiked at negative, 1 × LoD, and 2 × LoD concentrations with the characterized reference materials. For repeatability, each concentration was tested in eight replicates per day over three days by a single operator using a single instrument and a single reagent lot in one laboratory, resulting in 24 measurements per concentration level for each analyte.

Reproducibility was evaluated in three different laboratories, involving a total of three operators, three instruments, and three reagent lots. Each laboratory performed testing on a different testing day, using its own operator and instrument and a different reagent lot (Laboratory 1: operator 1, instrument 1, reagent lot 1, day 1; Laboratory 2: operator 2, instrument 2, reagent lot 2, day 2; Laboratory 3: operator 3, instrument 3, reagent lot 3, day 3). At each laboratory, each concentration level was tested in eight replicates, yielding a total of 24 replicates per concentration level for each analyte across the three laboratories. For each analyte and concentration level, the arithmetic mean, sample standard deviation, and coefficient of variation (CV%) of valid Cq results were calculated. The total CV was used as the principal precision measure, with a predefined acceptance criterion of CV ≤ 5%.

Analytical accuracy was evaluated using 72 classifications per analyte derived from the reproducibility measurement set, comprising 24 positive samples at 1 × LoD, 24 positive samples at 2 × LoD, and 24 negative samples. The positive and negative classifications were defined according to the prepared analytical status of the samples, and results were classified as true positive (TP), false positive (FP), true negative (TN), or false negative (FN) accordingly. Overall analytical accuracy was calculated as:

Accuracy (%) = 100 × [(TP + TN)/(TP + TN + FP + FN)]

Cohen’s kappa coefficient was also calculated to assess agreement between the observed assay classifications and the prepared analytical status. Total analytical accuracy and Cohen’s kappa were calculated using standard diagnostic performance methods in accordance with internationally accepted laboratory validation guidance [11,12,14]. Predefined acceptance criteria were an analytical accuracy of ≥95% and a Cohen’s kappa coefficient > 0.80.

### 2.11. Clinical Performance Evaluation and Specimen Selection

To evaluate the automated platform in a clinical setting, a single-center retrospective clinical agreement study was conducted at Medipol Mega University Hospital using archived, anonymized residual CSF specimens originally collected from patients undergoing routine investigation for suspected meningitis or encephalitis.

The source repository comprised 500 unique archived CSF specimens, including 250 specimens previously classified as positive for at least one pathogen included in the BS-MEP panel and 250 specimens classified as negative for all panel targets by routine comparator testing. Positive specimens were distributed across the individual analytes according to the availability of archived comparator-positive material. Clinical performance was evaluated using analyte-specific datasets of 250 specimens. For each analyte, all available comparator-positive specimens for that target were combined with comparator-negative specimens selected from the archived repository to produce a total analyte-specific denominator of 250. A specimen positive for one panel pathogen could be classified as negative for another pathogen and could therefore contribute to more than one analyte-specific evaluation. Consequently, analyte-specific denominators were not summed across targets.

Specimens were selected retrospectively using a non-consecutive, target-enriched sampling strategy to ensure representation of all panel analytes. Inclusion criteria were an archived CSF volume of ≥500 µL, preserved physical integrity, documented storage at −80 °C, no documented repeated freeze–thaw cycles, availability of an appropriate comparator result, anonymization, and a valid human RNase P internal-control signal.

Specimens were excluded when the available volume was <500 µL; when leakage, spillage, contamination, severe hemolysis, or other evidence of compromised integrity was present; when storage records were incomplete or multiple freeze–thaw cycles had occurred; when the internal control failed; when comparator information was unavailable; when anonymization requirements were not met; or when the specimen type was not CSF.

Samples were thawed under controlled conditions and analyzed after a single thaw cycle.

### 2.12. Comparator Methods and Result Classification

Routine results obtained before specimen archiving were used as the prespecified comparator classifications. Because no single reference test covers all 14 pathogens included in the BS-MEP panel, comparator methods varied by pathogen group. Bacterial and viral targets were classified using the CE-IVD-marked Seegene Allplex Meningitis-B, Meningitis-V1, and Meningitis-V2 assays, performed according to the manufacturers’ instructions. For the *Cryptococcus neoformans*/*gattii* complex, which is not included in these commercial molecular panels, fungal culture followed by species-level identification using the Bruker MALDI Biotyper Microflex LT/SH IVD system (Bruker Daltonik GmbH, Bremen, Germany) with IVD Library version 4.1.100 was used as the comparator method.

Archived specimens were not retested using the comparator methods after retrieval, and discordant investigational and comparator results were not adjudicated using a third independent method. Final classifications were therefore assigned relative to the prespecified routine comparator results. An investigational-positive/comparator-positive result was classified as true positive (TP); an investigational-negative/comparator-positive result as false negative (FN); an investigational-positive/comparator-negative result as false positive (FP); and an investigational-negative/comparator-negative result as true negative (TN).

Positive percent agreement (PPA) and negative percent agreement (NPA) were used as the principal agreement measures because the investigational assay was evaluated against routine comparator methods rather than against a single definitive reference standard. Positive predictive value (PPV) and negative predictive value (NPV) were also calculated.

### 2.13. Invalid Results and Repeat Testing

Two of 500 specimens initially failed to produce a valid human RNase P internal-control signal and were classified as invalid. Both specimens were retested according to the investigational assay instructions for use and produced valid results on repeat testing. The initial invalid/repeat rate was therefore 0.4%, and the valid repeat results were included in the final analysis.

### 2.14. Ethical Consideration

This clinical evaluation phase of this study was conducted in strict accordance with the ethical principles outlined in the Declaration of Helsinki. The study protocol was reviewed and approved by the Istanbul Medipol University Non-Interventional Clinical Research Ethics Committee (Decision No: 102, Document Ref: E-10840098-202.3.02-734; Date of Approval: 18 January 2024). Since this diagnostic evaluation relied entirely on archived, fully anonymized residual CSF specimens that were completely stripped of patient identifiers prior to analysis, the requirement for prospective individual informed consent was waived by the ethics committee.

### 2.15. Assumptions and Statistical Analysis

Statistical analyses were performed using Python 3.10.20 [11,13]. Data organization, validation, and construction of analyte-specific contingency tables were conducted using pandas 2.3.3. Numerical calculations and bootstrap resampling were performed using NumPy 2.2.6. Standard-normal probability calculations were conducted using SciPy 1.15.3. Probit regression and exact binomial confidence intervals were implemented using statsmodels 0.14.6, and Cohen’s kappa coefficient was calculated using scikit-learn 1.7.2.

For LoD estimation, replicate results at each analyte concentration were coded as binary outcomes, with detected results coded as 1 and non-detected results coded as 0. Analyte concentrations were log10-transformed and modeled using statsmodels.api.GLM with a binomial error distribution and Probit link function, implemented using statsmodels.genmod.families.Binomial and statsmodels.genmod.families.links.Probit. The fitted model was defined as:

Φ^−1^[p(x)] = β_0_ + β_1_log_10_(x),

where p(x) represents the probability of detection at analyte concentration x, Φ^−1^ represents the inverse standard-normal cumulative distribution function, and β_0_ and β_1_ are the fitted regression coefficients.

The standard-normal quantile corresponding to a detection probability of 0.95 was calculated using scipy.stats.norm.ppf(0.95). LoD95 was obtained by inverse prediction:

log_10_(LoD95) = [Φ^−1^(0.95) − β_0_]/β_1_.

The estimated LoD concentration was subsequently evaluated using 24 replicates. The hit rate was calculated as the number of positive replicates divided by the total number tested. The predefined verification criterion was ≥23/24 positive replicates, corresponding to an observed hit rate of ≥95.8%.

Precision was evaluated using Cq measurements obtained at 1 × LoD and 2 × LoD. For each analyte and concentration, the arithmetic mean and sample standard deviation were calculated using numpy.mean and numpy.std(ddof = 1), respectively. CV was calculated as:

CV (%) = 100 × SD(Cq)/mean(Cq).

Total CV was calculated from all valid Cq measurements generated under the predefined repeatability and reproducibility conditions.

Analytical accuracy and clinical diagnostic performance were evaluated using 2 × 2 contingency tables constructed with pandas.crosstab. Results were classified as TP, FP, TN, or FN. Performance measures were calculated as follows:

PPA = TP/(TP + FN);

NPA = TN/(TN + FP);

PPV = TP/(TP + FP);

NPV = TN/(TN + FN);

overall accuracy = (TP + TN)/(TP + TN + FP + FN).

PPA and NPA were used as the primary agreement terminology because the investigational assay was evaluated against comparator methods rather than against a single definitive reference standard.

Two-sided 95% confidence intervals for PPA, NPA, PPV, NPV, and overall accuracy were calculated using the Clopper–Pearson exact binomial method, implemented with statsmodels.stats.proportion.proportion_confint, alpha = 0.05, and method = “beta”.

Agreement beyond chance was assessed using the unweighted Cohen’s kappa coefficient calculated with sklearn.metrics.cohen_kappa_score and weights = None. Kappa was defined as:

κ = (P_o_ − P_e_)/(1 − P_e_),

where P_o_ is the observed proportion of agreement and P_e_ is the agreement expected by chance from the marginal distributions.

The 95% confidence interval for the pooled kappa estimate was obtained by nonparametric bootstrap resampling of paired investigational and comparator results. Bootstrap resampling was performed using numpy.random.default_rng(seed = 42). A total of 10,000 bootstrap samples, each equal in size to the original dataset and sampled with replacement, were generated. Cohen’s kappa was recalculated for each sample, and the 2.5th and 97.5th percentiles calculated using numpy.percentile were reported as the two-sided confidence limits.

Analyses were conducted separately for each analyte and, where appropriate, for the pooled clinical dataset. Because this was an analytical and clinical performance-validation study, analyses were primarily descriptive, and no adjustment for multiple hypothesis testing was applied.

Acceptance criteria were predefined before data analysis. A model-estimated detection probability of 95% was used for LoD estimation, and ≥23 positive results among 24 replicates were required for verification. CV ≤ 5% and analytical accuracy ≥ 95% were predefined as stringent, risk-based internal protocol criteria for acceptable reproducibility and qualitative classification. These thresholds were not interpreted as universal CLSI-mandated limits. Cohen’s kappa > 0.80 was required to demonstrate strong agreement beyond chance.

## 3. Results

### 3.1. Limit of Detection

The LoD values for CSF samples ranged from 472 to 2118 genome copies/mL (Table 2). *Escherichia coli* K1 exhibited the lowest LoD (472 copies/mL), while human parechovirus had the highest (2118 copies/mL). LoD values clustered below 1600 copies/mL for most bacterial targets and between 1000 and 2100 copies/mL for viral targets. All analytes met the predefined LoD verification criterion of ≥23/24 positive replicates at the manufacturer-claimed concentrations.

### 3.2. Inclusivity and In-Silico Inclusivity

All strains tested at the LoD concentration were detected in five of five replicates (100% detection). Mean Cq values ranged from 24.92 cycles (*Listeria monocytogenes*) to 26.48 cycles (*Streptococcus pneumoniae*), with standard deviations < 0.5 cycles.

In silico sequence analysis demonstrated high coverage across the compiled reference databases when allowing up to one mismatch, with further improvement up to two mismatches permitted. Localized reductions in alignment matches were noted for the *Streptococcus agalactiae* forward primer (77.29% at 0 mismatch) and the human parechovirus (34.68% at 0 mismatch). However, these isolated primer mismatches did not imply reduced assay performance, as successful target detection is determined by the combined performance of the complete primer–probe set. Lower perfect-match percentages therefore identify regions requiring continued sequence surveillance but do not, by themselves, establish reduced analytical performance (Table 3).

### 3.3. Cross-Reactivity

No cross-reactivity was observed. All non-target organisms yielded negative results at a concentration of 10^5^ copies/mL.

### 3.4. Precision and Accuracy

The CV % at 1 × LoD ranged from 0.86% (HSV-1) to 1.38% (human parechovirus), and at 2 × LoD from 0.86% (*Haemophilus influenzae*) to 1.19% (*Listeria monocytogenes*), satisfying the acceptance criterion (CV ≤ 5%). Total accuracy exceeded 97% for all analytes, reaching 100% for seven of fourteen targets (*Neisseria meningitidis*, *Haemophilus influenzae*, *Streptococcus agalactiae*, HSV-2, HHV-6, human parechovirus and *E. coli* K1). Cytomegalovirus had the lowest accuracy (95.83%) but still met the ≥95% criterion. Combined classification analytical accuracy calculations surpassed 97% across all 14 targets, achieving absolute 100% precision for seven analytes (*N. meningitidis*, *H. influenzae*, *S. agalactiae*, HSV-2, HHV-6, human parechovirus, and *E. coli* K1). Cohen’s kappa values ranged from 0.977 to 0.978, indicating almost perfect agreement.

### 3.5. Clinical Performance

In a separate pooled specimen-level analysis of the 500 unique archived CSF specimens, 243 of 250 comparator-positive specimens were detected by the investigational assay, while 7 were not detected; all 250 comparator-negative specimens remained negative. Pooled positive percent agreement (PPA) was 97.2% (95% CI, 94.3–98.9%), while negative percent agreement (NPA) was 100% (95% CI, 98.5–100%). Positive predictive value (PPV) was 100% (95% CI, 98.5–100%), negative predictive value (NPV) was 97.3% (95% CI, 94.5–98.9%), and overall accuracy was 98.6% (95% CI, 97.1–99.4%). Agreement at the pooled specimen level was strong, with a Cohen’s κ of 0.972 (bootstrap 95% CI, 0.948–0.992).

At the individual analyte level, PPA ranged from 90.9% to 100%, with the false-negative classifications observed for the *Cryptococcus neoformans*/*gattii* complex, *Neisseria meningitidis*, *Streptococcus agalactiae*, cytomegalovirus, enterovirus, human parechovirus, and human herpesvirus 6 (Table 4). NPA and PPV were 100% across all analytes, with no false-positive classifications observed.

## 4. Discussion

The comprehensive performance evaluation conducted in this study demonstrates that the 14-target multiplex panel meets rigorous analytical and clinical criteria when operated within a fully automated workflow. The mathematical limits of detection calculated across all bacterial, viral, and fungal targets are highly comparable to, or show improvements over, previously published manual syndromic validation data [19]. Furthermore, the wet-lab target-detection verification confirmed consistent detection of the characterized reference materials at the established LoD, while the in silico analysis provided broader sequence-level assessment across curated taxon-specific datasets. In the clinical evaluation, the investigational assay showed high agreement with the prespecified routine comparator classifications, including 100% NPA and no observed false-positive classifications. Our findings are consistent with previous evaluations of multiplex molecular panels for meningitis and encephalitis, which have generally reported sensitivities exceeding 90% and specificities approaching or reaching 100% for most target pathogens. Systematic reviews have further demonstrated that syndromic molecular testing enables earlier pathogen identification, facilitates timely optimization of antimicrobial therapy, and improves the overall diagnostic approach to suspected central nervous system infections [20]. The diagnostic performance observed in the present study aligns well with this growing body of evidence while extending the available literature by evaluating a fully automated high-throughput workflow with continuous specimen loading capabilities.

In severe CNS infections, high diagnostic specificity is paramount [21]. Early and accurate pathogen detection is critical for timely initiation of targeted therapy, which can directly impact prognosis and reduce the incidence of long-term sequelae in infections with potentially devastating outcomes [22]. False-positive results frequently trigger a cascading sequence of clinical missteps, including prolonged hospitalizations, unnecessary exposure to potentially toxic broad-spectrum antimicrobials, and invasive diagnostic follow-ups like serial lumbar punctures.

The absence of false-positive classifications resulted in a PPV of 100% in the evaluated study population; however, this estimate should be interpreted in the context of the target-enriched study design and should not be extrapolated directly to population-level predictive performance. Concurrently, the small subset of single false-negative classifications emphasizes that standalone molecular findings should always be cross-referenced with clinical history, CSF biochemistry (glucose and protein ratios), and specialized local imaging data.

The ability to detect a wide spectrum of bacterial and viral pathogens—including high-risk agents such as *Neisseria meningitidis*, *Streptococcus pneumoniae*, *Listeria monocytogenes*, enteroviruses, and HSV—in a single CSF sample enhances clinical utility, particularly in settings where prompt decision-making is essential.

A major programmatic strength of this diagnostic validation is the integration of the *Cryptococcus neoformans*/*gattii* complex directly within the core testing strip. Cryptococcal meningoencephalitis remains an insidious cause of high mortality, particularly among immunocompromised patients [23,24]. Because multiplex PCR reference assays rarely bundle fungal markers alongside basic bacterial targets, our design relied on definitive species identification via automated MALDI-TOF mass spectrometry as the clinical benchmark. In the clinical evaluation, 9 of 10 comparator-positive specimens were detected, corresponding to a PPA of 90.9%; this estimate should be interpreted cautiously because of the small number of comparator-positive specimens for this target.

Beyond analytical accuracy, laboratory workflow has become an increasingly important determinant of the overall clinical value of molecular diagnostic platforms. Although several commercially available multiplex PCR systems demonstrate excellent diagnostic performance, many are primarily optimized for single-sample or low-volume testing, making them particularly suitable for emergency departments or near-patient applications. In contrast, reference microbiology laboratories often process large numbers of specimens daily and therefore require automated systems capable of maintaining rapid turnaround times without compromising throughput or standardization [25]. The continuous-loading workflow evaluated in the present study addresses this operational challenge by permitting additional specimens to be introduced during active processing, thereby minimizing dependence on batch testing while maintaining fully automated sample preparation, nucleic acid extraction, amplification, and result interpretation. Such workflow characteristics have the potential to improve laboratory efficiency while preserving the rapid reporting required for time-sensitive central nervous system infections [26].

The operational characteristics of the evaluated platform are also relevant to its potential use in clinical laboratories. The system integrates automated sample preparation, nucleic acid extraction, amplification, and result interpretation, with an approximately 80-min analytical turnaround and continuous specimen loading. These characteristics may facilitate flexible laboratory workflow compared with strictly batch-based processing. However, workflow efficiency, hands-on time, cost-effectiveness, and the effect of continuous loading on clinical turnaround were not formally evaluated in this study and therefore require prospective assessment in routine laboratory settings.

Our study has several limitations that need to be addressed. First, the limits of detection were derived from controlled, spiked negative matrices, which may not fully reflect the natural biological variability, cellular debris, or varying viscosity encountered in real-world patient specimens. Second, while total in silico coverage was highly acceptable, the forward primers for *Streptococcus agalactiae* and human parechovirus exhibited localized sequence mismatches that require continuous bioinformatic monitoring. Third, endogenous and exogenous physicochemical interferents were not empirically evaluated in the wet laboratory. Although heavy blood contamination, chlorhexidine, or ethanol residues were identified as potential interferents, other factors such as elevated protein concentrations, increased leukocyte counts, xanthochromia, or additional PCR inhibitory substances may also reduce nucleic acid extraction efficiency or amplification performance under certain clinical conditions [27]. While modern extraction chemistries are designed to minimize many inhibitory effects, future studies should systematically investigate these variables using standardized interference protocols and evaluate approaches such as inhibitor-resistant polymerases and optimized extraction workflows to further enhance the robustness of multiplex RT-qPCR assays.

The clinical evaluation was retrospective and based on archived residual CSF specimens selected using a non-consecutive, target-enriched strategy to ensure representation of all panel analytes. Consequently, the analyte-specific datasets did not reflect the natural prevalence of CNS pathogens in routine clinical practice, and the observed PPV and NPV should not be interpreted as population-level predictive values. Comparator classifications were based on routine results obtained before specimen archiving; archived specimens were not retested using the comparator methods, and discordant results were not adjudicated using a third independent method. Therefore, agreement estimates are relative to the prespecified routine comparator classifications and may be influenced by limitations or misclassification of the comparator methods. In addition, the limited number of comparator-positive specimens for several low-prevalence analytes resulted in wider confidence intervals for some analyte-level PPA estimates. Finally, prolonged storage and potential freeze–thaw exposure may have affected nucleic acid integrity in some archived specimens and could have contributed to some false-negative results. Prospective multicenter studies using consecutive clinical specimens and standardized interference and discrepancy-resolution protocols are warranted to further validate the assay under routine clinical conditions.

## 5. Conclusions

The Bioeksen Meningitis/Encephalitis Panel performed on the automated Sigmoida Lab platform demonstrated strong analytical performance and high comparative agreement with the prespecified routine comparator classifications across the evaluated 14-target panel. The assay showed low analytical detection thresholds, reproducible Cq measurements, consistent detection of the characterized reference materials at the established LoD, broad sequence-level coverage in the in silico analysis, and no observed cross-reactivity in the evaluated non-target panel. The clinical evaluation showed high agreement with routine comparator classifications, including 100% negative percent agreement and no observed false-positive classifications. The automated workflow and continuous-loading capability are relevant platform characteristics for further evaluation in routine laboratory settings. However, the retrospective, non-consecutive, target-enriched study design does not establish clinical outcomes, treatment impact, cost-effectiveness, or superiority over conventional diagnostic workflows. Prospective multicenter studies using consecutive clinical specimens and standardized discrepancy-resolution procedures are warranted to further evaluate clinical performance and real-world clinical utility.

## Figures and Tables

**Table 1:** Molecular targets and expected amplicon lengths of the BS-MEP assay.

Target Pathogen	Nucleic Acid Type	Target Gene or Genomic Region	Expected Amplicon Length
*Cryptococcus neoformans/gattii* complex	DNA	5.8S rRNA	134 bp
*Listeria monocytogenes*	DNA	*hly*	86 bp
*Neisseria meningitidis*	DNA	*sodC*	287 bp
*Streptococcus pneumoniae*	DNA	*lytA*	202 bp
*Haemophilus influenzae*	DNA	*glpQ*	151 bp
*Streptococcus agalactiae*	DNA	*cfb*	248 bp
*Escherichia coli* K1	DNA	*neuC*	168 bp
Cytomegalovirus	DNA	UL54	176 bp
Enterovirus	RNA	5′ untranslated region	142 bp
Human parechovirus	RNA	5′ untranslated region	194 bp
Varicella-zoster virus	DNA	ORF71	155 bp
Herpes simplex virus 1	DNA	US6	77 bp
Herpes simplex virus 2	DNA	US4	111 bp
Human herpesvirus 6	DNA	NEC2	127 bp

**Table 2:** Analytical Performance and Limit of Detection (LoD) Summary.

Target Pathogen	Verified LoD (Genome Copies/mL)	Inclusivity Cq Avg (Cycles)	Inclusivity SD (Cycles)	Hit Rate	CV% at 1 × LoD	CV% at 2 × LoD	Total Analytical Accuracy (%)	Cohen’s Kappa (κ)
*Escherichia coli* K1	472	25.59	0.18	5/5	0.87	1.05	100.00	0.978
*Haemophilus influenzae*	696	26.26	0.13	5/5	1.02	0.86	100.00	0.978
*Listeria monocytogenes*	702	24.92	0.24	5/5	1.01	1.19	98.61	0.978
*Cryptococcus gattii*	929	26.06	0.22	5/5	1.10	1.03	98.61	0.977
*Herpes simplex virus 1*	1009	25.54	0.19	5/5	0.86	0.98	98.61	0.977
*Neisseria meningitidis*	1306	26.05	0.34	5/5	0.98	0.96	100.00	0.978
*Human herpesvirus 6*	1445	25.27	0.16	5/5	1.16	0.94	100.00	0.978
*Varicella-zoster virus*	1445	26.40	0.21	5/5	0.88	1.08	97.22	0.978
*Streptococcus pneumoniae*	1563	26.48	0.37	5/5	0.90	1.15	98.61	0.978
*Cryptococcus neoformans*	1653	26.06	0.22	5/5	1.10	1.03	98.61	0.977
*Herpes simplex virus 2*	1761	25.17	0.44	5/5	0.93	0.90	100.00	0.978
*Streptococcus agalactiae*	2073	26.26	0.13	5/5	0.87	1.03	100.00	0.978
*Cytomegalovirus*	2088	25.73	0.41	5/5	0.92	1.01	95.83	0.977
*Enterovirus*	2107	26.38	0.32	5/5	0.98	1.08	97.22	0.978
*Human parechovirus*	2118	25.94	0.38	5/5	1.38	1.04	100.00	0.978

**Table 3:** In Silico Inclusivity analysis showing the percentage of target sequences exhibiting zero, one, or two mismatches with the forward primer, reverse primer, and probe for each analyte. Lower perfect-match percentages for individual primers do not necessarily indicate reduced analytical detection, as assay performance depends on the combined performance of the complete primer–probe set.

Analyte Target	Forward 0	Forward 1	Forward 2	Reverse 0	Reverse 1	Reverse 2	Probe 0	Probe 1	Probe 2
*Listeria monocytogenes*	99.79	0.21	0.00	99.03	0.54	0.43	99.36	0.64	0.00
*Neisseria meningitidis*	100.00	0.00	0.00	100.00	0.00	0.00	98.75	1.25	0.00
*Streptococcus pneumoniae*	100.00	0.00	0.00	100.00	0.00	0.00	100.00	0.00	0.00
*Haemophilus influenzae*	100.00	0.00	0.00	97.86	2.14	0.00	99.29	0.71	0.00
*Streptococcus agalactiae*	77.29	22.71	0.00	100.00	0.00	0.00	100.00	0.00	0.00
*Varicella-zoster virus*	99.29	0.71	0.00	99.65	0.35	0.00	98.94	1.06	0.00
*Herpes simplex virus 1*	99.39	0.61	0.00	99.39	0.61	0.00	100.00	0.00	0.00
*Herpes simplex virus 2*	98.16	0.91	0.73	98.16	1.10	0.00	99.26	0.73	0.00
*Human herpesvirus 6*	100.00	0.00	0.00	100.00	0.00	0.00	99.31	0.69	0.00
*Cryptococcus complex*	99.47	0.52	0.00	87.76	12.23	0.00	98.41	0.53	1.06
*Enterovirus*	94.32	4.78	0.54	96.22	3.35	0.35	96.99	2.83	0.06
*Human parechovirus*	34.68	63.71	0.81	75.81	23.39	0.81	96.77	3.23	0.00
*Escherichia coli* K1	95.65	4.35	0.00	100.00	0.00	0.00	100.00	0.00	0.00

**Table 4:** Clinical Performance of the Bioeksen Meningitis/Encephalitis Panel. Clinical performance was evaluated separately for each analyte using a target-specific dataset of 250 specimens derived from the overall cohort of 500 unique archived CSF specimens; therefore, analyte-specific datasets may include overlapping specimens. Values are presented as point estimates with two-sided 95% confidence intervals calculated using the Clopper–Pearson exact method. PPA, positive percent agreement; NPA, negative percent agreement; PPV, positive predictive value; NPV, negative predictive value.

Pathogen Target	TP	FP	TN	FN	PPA, % (95% CI)	NPA, % (95% CI)	PPV, % (95% CI)	NPV, % (95% CI)
*Cryptococcus neoformans*/*gattii* complex	10	0	239	1	90.9 (58.7–99.8)	100.0 (98.5–100.0)	100.0 (69.2–100.0)	99.6 (97.7–100.0)
*Listeria monocytogenes*	10	0	240	0	100.0 (69.2–100.0)	100.0 (98.5–100.0)	100.0 (69.2–100.0)	100.0 (98.5–100.0)
*Neisseria meningitidis*	30	0	219	1	97.0 (83.3–99.9)	100.0 (98.3–100.0)	100.0 (88.4–100.0)	99.5 (97.5–100.0)
*Streptococcus pneumoniae*	30	0	220	0	100.0 (88.4–100.0)	100.0 (98.3–100.0)	100.0 (88.4–100.0)	100.0 (98.3–100.0)
*Haemophilus influenzae*	25	0	225	0	100.0 (86.3–100.0)	100.0 (98.4–100.0)	100.0 (86.3–100.0)	100.0 (98.4–100.0)
*Streptococcus agalactiae*	25	0	224	1	96.2 (80.4–99.9)	100.0 (98.4–100.0)	100.0 (86.3–100.0)	99.6 (97.5–100.0)
*Escherichia coli* K1	25	0	225	0	100.0 (86.3–100.0)	100.0 (98.4–100.0)	100.0 (86.3–100.0)	100.0 (98.4–100.0)
Cytomegalovirus	10	0	239	1	90.9 (58.7–99.8)	100.0 (98.5–100.0)	100.0 (69.2–100.0)	99.6 (97.7–100.0)
Enterovirus	25	0	224	1	96.2 (80.4–99.9)	100.0 (98.4–100.0)	100.0 (86.3–100.0)	99.6 (97.5–100.0)
Human parechovirus	10	0	239	1	90.9 (58.7–99.8)	100.0 (98.5–100.0)	100.0 (69.2–100.0)	99.6 (97.7–100.0)
Varicella-zoster virus	10	0	240	0	100.0 (69.2–100.0)	100.0 (98.5–100.0)	100.0 (69.2–100.0)	100.0 (98.5–100.0)
Herpes simplex virus 1	10	0	240	0	100.0 (69.2–100.0)	100.0 (98.5–100.0)	100.0 (69.2–100.0)	100.0 (98.5–100.0)
Herpes simplex virus 2	10	0	240	0	100.0 (69.2–100.0)	100.0 (98.5–100.0)	100.0 (69.2–100.0)	100.0 (98.5–100.0)
Human herpesvirus 6	10	0	239	1	90.9 (58.7–99.8)	100.0 (98.5–100.0)	100.0 (69.2–100.0)	99.6 (97.7–100.0)

## Data Availability

The anonymous datasets analyzed during the current study are not publicly available due to ethical restrictions and institutional data protection policies. However, the data are available from the corresponding author upon reasonable request.

## Supplementary Materials

The following supporting information can be downloaded at: https://www.mdpi.com/article/10.3390/microorganisms14091927/s1, Supplementary Table S1. Reference materials used for analytical validation. Supplementary Table S2. Sequence datasets used for the in silico inclusivity analysis.
