# Supplementary material for: Analytical Performance and Clinical Evaluation of a Fully Automated Multiplex RT-qPCR Platform for Rapid Detection of Central Nervous System Pathogens
Source: Microorganisms. 2026 Sep 1;14(9):1927. doi: 10.3390/microorganisms14091927 (PMC13609735; doi:10.3390/microorganisms14091927)
Supplement: Supplementary file 1 [file microorganisms-14-01927-s001.zip › microorganisms-4381691-supplementary.pdf]

Supplementary Table S1. Reference materials used for analytical validation

A. Target reference materials

Target/organism	Catalog number	Analytical use
Cryptococcus neoformans	NR-CN-C	LoD determination; wet-lab target-detection verification; precision; analytical accuracy
Cryptococcus gattii	NR-CGA-C	LoD determination; wet-lab target-detection verification; precision; analytical accuracy
Neisseria meningitidis	NR-NM-C	LoD determination; wet-lab target-detection verification; precision; analytical accuracy
Streptococcus pneumoniae	NR-STP-C	LoD determination; wet-lab target-detection verification; precision; analytical accuracy
Haemophilus influenzae	NR-HI-C	LoD determination; wet-lab target-detection verification; precision; analytical accuracy
Streptococcus agalactiae	NR-STA-C	LoD determination; wet-lab target-detection verification; precision; analytical accuracy
Herpes simplex virus 1	NR-HSV1-C	LoD determination; wet-lab target-detection verification; precision; analytical accuracy
Herpes simplex virus 2	NR-HSV2-C	LoD determination; wet-lab target-detection verification; precision; analytical accuracy
Human herpesvirus 6	NR-HV6-C	LoD determination; wet-lab target-detection verification; precision; analytical accuracy
Enterovirus	NR-HEV-C	LoD determination; wet-lab target-detection verification; precision; analytical accuracy
Human parechovirus	NR-HPA-C	LoD determination; wet-lab target-detection verification; precision; analytical accuracy
Escherichia coli K1	NR-ECK-C	LoD determination; wet-lab target-detection verification; precision; analytical accuracy
Varicella-zoster virus	NR-VZV-C	LoD determination; wet-lab target-detection verification; precision; analytical accuracy
Cytomegalovirus	NR-CMV-C	LoD determination; wet-lab target-detection verification; precision; analytical accuracy
Listeria monocytogenes	NR-LM-C	LoD determination; wet-lab target-detection verification; precision; analytical accuracy

B. Wet-lab exclusivity panel

**Non-target organism**	**Catalog number**
Candida albicans	NR-CA-S
Candida glabrata	NR-CG-S
Candida krusei	NR-CK-S
Cryptococcus albidus	NR-CAL-S
Cryptococcus laurentii	NR-CRL-S
Saccharomyces cerevisiae	NR-SAC-S
Epstein–Barr virus	NR-EPV-S
Streptococcus anginosus	NR-STN-S
Streptococcus dysgalactiae	NR-SD-S
Streptococcus intermedius	NR-SIN-S
Streptococcus mitis	NR-SMI-S
Streptococcus oralis	NR-SO-S
Streptococcus sanguinis	NR-STS-S
Citrobacter freundii	Not specified
Corynebacterium striatum	Not specified
Enterobacter aerogenes	Not specified
non-K1 *Escherichia coli*	Not specified
Haemophilus ducreyi	Not specified

Supplementary Table S2. Sequence datasets used for the in silico inclusivity analysis

Target taxon	Final unique sequences	Download date	Database
Cytomegalovirus	440	28 October 2024	NCBI Virus
Human parechovirus	124	28 October 2024	NCBI Virus
Enterovirus	4,810	28 October 2024	NCBI Virus
Varicella-zoster virus	564	28 October 2024	NCBI Virus
Herpes simplex virus 1	537	29 January 2025	NCBI Virus
Herpes simplex virus 2	27	28 October 2024	NCBI Virus
Human herpesvirus 6	290	29 January 2025	NCBI Virus
Haemophilus influenzae	142	4 November 2024	NCBI Nucleotide
Streptococcus pneumoniae	320	4 November 2024	NCBI Nucleotide
Neisseria meningitidis	161	29 January 2025	NCBI Nucleotide
Listeria monocytogenes	931	29 January 2025	NCBI Nucleotide
Streptococcus agalactiae	229	4 November 2024	NCBI Nucleotide
Escherichia coli K1	253	4 November 2024	NCBI Nucleotide
Cryptococcus neoformans/gattii complex	192	4 November 2024	NCBI Nucleotide
